# CSF and blood biomarkers in amyotrophic lateral sclerosis: protocol for a systematic review and meta-analysis

**DOI:** 10.1186/s13643-018-0913-4

**Published:** 2018-12-20

**Authors:** Elmira Agah, Fatemeh Saleh, Hossein Sanjari Moghaddam, Amene Saghazadeh, Abbas Tafakhori, Nima Rezaei

**Affiliations:** 1 NeuroImmunology Research Association (NIRA), Universal Scientific Education and Research Network (USERN), Tehran, Iran; 2 Students’ Scientific Research Center, Tehran University of Medical Sciences, Tehran, Iran; 3 School of Medicine, Tehran University of Medical Sciences, Tehran, Iran; 4 Research Center for Immunodeficiencies (RCID), Children’s Medical Center, Tehran University of Medical Sciences, Tehran, Iran; 5 Systematic Review and Meta-analysis Expert Group (SRMEG), Universal Scientific Education and Research Network (USERN), Boston, MA USA; 6 Iranian Center of Neurological Research (ICNR), Neuroscience Institute, Tehran University of Medical Sciences, Tehran, Iran; 7 Department of Immunology, School of Medicine, Tehran University of Medical Sciences, Tehran, Iran

**Keywords:** Amyotrophic lateral sclerosis, ALS, Neurodegeneration, Biomarker, Biological marker

## Abstract

**Background:**

Amyotrophic lateral sclerosis (ALS) is a highly progressive and debilitating neurodegenerative disease, which usually leads to the death of affected individuals within a few years after the onset of symptoms. ALS is currently incurable and very little is known about its pathophysiology. Finding validated biomarkers will help us to advance our understanding of ALS etiology and find better strategies for early diagnosis and management of the disease. The main aim of the present systematic review is to evaluate the concentration of 11 frequently reported biomarkers for ALS in peripheral blood and CSF of patients diagnosed with ALS compared with controls.

**Methods:**

This systematic review protocol has been established according to the Preferred Reporting Items for Systematic Reviews and Meta-Analyses Protocol (PRISMA-P) 2015 guideline. We will include all types of observational studies with human subjects that investigated the concentrations of intended biomarkers (amyloid beta (Aβ-42), tau and phosphorylated tau (p-Tau), neurofilaments, S100β, cystatin C, progranulin (PGRN), glial fibrillary acidic protein (GFAP), monocyte chemoattractant protein-1 (MCP-1), brain-derived neurotrophic factor (BDNF), TAR DNA-binding protein-43 (TDP43), YKL-40, and CHIT1 in CSF or peripheral blood of ALS patients for initial assessment. Also, we will include case series with a minimum of 10 cases and clinical trials which have measured baseline biomarker levels. Case studies, case reports, reviews, letters, and animal and in vitro studies will be excluded. Multiple electronic databases including Cochrane Library, MEDLINE (PubMed), ISI Web of Science, and EMBASE will be searched to find all eligible articles published since 1980. No language restriction will be applied. All titles and abstracts retrieved by searching information sources will be evaluated independently by two authors against the eligibility criteria. The following information will be extracted from each included study by two independent authors: bibliographic details (first author, study title, year of publication, country), demographics and clinical information (number of patients and controls, type of ALS and controls, study design, age, gender, specimen, biomarkers levels, ALS functional rating scale Revised (ALSFRS-R), duration of disease), and measurements (method, value type, biomarkers levels). We will use the extracted mean and standard deviation (SD) of biomarkers concentrations to calculate the standardized mean difference (SMD) and 95% confidence intervals (CI). The primary outcome measures are the mean difference of biomarker levels between ALS patients and controls, different types of ALS, and ALS patients with genetic mutations.

**Discussion:**

We will systematically review the literature and analyze studies of biomarker level in CSF and peripheral blood of patients with ALS and controls. The results will help us to identify biomarkers with possible diagnostic and prognostic value.

**Systematic review registration:**

PROSPERO CRD42017078127

**Electronic supplementary material:**

The online version of this article (10.1186/s13643-018-0913-4) contains supplementary material, which is available to authorized users.

## Background

Amyotrophic lateral sclerosis (ALS) is a highly progressive neurodegenerative disease, which usually leads to severe disability, respiratory dysfunction, and death in the affected patients within 3 to 5 years after the onset of symptoms [1]. Moreover, ALS is the most common type of motor neuron disease (MND).

ALS is relatively a rare disease with the worldwide incidence and prevalence rates of 2 and 6 per 100,000 respectively [2, 3]. However, while the population is getting older, it is estimated that the number of patients with ALS will significantly increase in the following 20 years mainly in developing countries such as Iran [4]. Despite the low incidence of ALS, its economic burden to society and patients is higher than other common neurological disorders such as stroke, dementia, and Parkinson’s disease [5]. ALS is more common among the elderly population, but it can be diagnosed in younger individuals too [6]. The overall incidence rate of ALS is higher in males than females [3], but bulbar-onset ALS is diagnosed slightly more often in women [7].

Clinically, ALS is a heterogeneous disease, and patients may present with different signs and symptoms such as progressive muscle weakness and atrophy, dysarthria and dysphagia, cognitive impairment, and executive dysfunction, based on the degree of upper and lower motor neuron involvement, site of onset (bulbar or spinal), and presence of genetic mutations [8]. Most ALS cases are sporadic with no apparent familial history of other related neurological conditions such as frontotemporal dementia (FTD). According to the population-based studies, less than 5% of ALS cases are familial, but this percentage may be over 10 in referral centers [9, 10]. Patients with the familial type of ALS are usually younger than sporadic patients, and ALS-related gene mutations are more frequent among them [11]. Patients with classic ALS or spinal onset ALS present with a combination of the upper motor neuron (UMN) and lower motor neuron (LMN) signs in their limbs. Bulbar muscles are the initial site of involvement in approximately one third of ALS cases. It is estimated that the incidence rate of bulbar-onset ALS is more than 40% in some European nations and is lower than 28% in North America and Asia [9]. Dysarthria, dysphagia, and tongue fasciculations are the hallmarks of bulbar-onset ALS [12]. A minority of patients may initially present with either of UMN or LMN signs and symptoms.

No specific diagnostic test is available for ALS, and patients with characteristic findings are usually diagnosed only when other possible causes were ruled out. According to the ALS diagnostic criteria, Revised EI Escorial criteria [13], patients are divided into groups with definite, probable, possible, and familial ALS based on the evidence available from clinical, laboratory, and electrodiagnostic investigations. Epidemiologic studies have shown that ALS patients are usually diagnosed with a 12-month delay, and around 10% of patients diagnosed with ALS are later recognized to have another similar disorder [14].

The exact etiology of ALS is currently unknown. In recent years, a growing body of literature has strongly supported the pathologic involvement of oxidative stress, mitochondrial dysfunction, neuroinflammation, infection, and glutamate toxicity in ALS [8]. Based on these presumed pathogenic mechanisms, various drugs were tested for possible clinical efficacy, but unfortunately, none of them were successful so far.

To advance our understanding of ALS etiology and find a better strategy for early diagnosis and management of the disease, validated biomarkers, which are defined as “objectively measured and evaluated parameters for indication of pathological processes, disease progression or response to pharmacological interventions,” are urgently needed [15]. Body fluids especially blood and cerebrospinal fluid (CSF) are best places for finding a biomarker. Blood samples are obtained easily by a minimally invasive procedure, but they are less sensitive than CSF for measurement of CNS-related markers. CSF has direct interaction with CNS and is a better choice for seeking biomarkers of neurological diseases. However, compared with blood, CSF is collected by a more invasive procedure, which might limit its applicability in the clinical setting.

## Objectives

The aim of the present systematic review is to evaluate the concentration of 11 frequently reported biomarkers for ALS in peripheral blood and CSF of patients diagnosed with ALS compared with controls.

## Methods

This systematic review protocol has been established according to the Preferred Reporting Items for Systematic Reviews and Meta-Analyses Protocol (PRISMA-P) 2015 guideline (Additional file 1).

## Eligibility criteria

### Type of studies

We will include all types of observational studies with human subjects that investigated the concentrations of intended biomarkers in CSF and peripheral blood of ALS patients versus controls (healthy participants or patients with other types of neurological disorders) for initial assessment. We may include studies that have no control group if they have reported biomarkers levels among different types of ALS patients or patients with and without specific gene mutations. Also, we will include case series with a minimum of 10 cases and clinical trials which have measured baseline biomarkers levels. Case studies, case reports, reviews, letters, and animal and in vitro studies will be excluded.

### Type of participants

Male and female adults (18 years of age or older) with ALS diagnosis according to El Escorial criteria (revised version or original based on the time of the study) will be included. We will exclude patients with neurological and non-neurological comorbidities that may affect biomarker levels.

### Types of interventions

Studies which evaluated the concentration of either of following biomarkers in CSF and peripheral blood of ALS patients and controls with appropriate measurement methods, e.g., enzyme-linked immunosorbent assay.

Biomarkers: Amyloid beta (Aβ-42); Tau and phosphorylated tau (p-Tau); Neurofilaments; S100β; Cystatin C; progranulin (PGRN); Glial fibrillary acidic protein (GFAP); Monocyte chemoattractant protein-1 (MCP-1); Brain-derived neurotrophic factor (BDNF); TAR DNA-binding protein-43 (TDP43); YKL-40; and chitotriosidase (CHIT1).

### Types of outcome measures

#### Primary outcomes

- Mean difference of biomarkers concentrations between ALS patients and controls

#### Secondary outcomes

- Mean difference of biomarker concentrations between male and female patients
- Association between biomarker concentrations and cognitive performance
- Association between biomarker concentrations and ALS progression rate
- Association between biomarker concentrations and ALS functional rating scale (ALSFRS)
- Association between biomarker concentrations and survival

### Information sources

#### Electronic search

We will search multiple electronic databases including Cochrane Library, MEDLINE (PubMed), ISI Web of Science, and EMBASE to find all eligible articles published since 1980. No language restriction will be applied. We will search these databases with the keywords listed in Additional file 2.

#### Searching other resources

The reference lists of all included articles and relevant reviews will be checked to find further titles. We will search conference papers and other grey literature via sources recommended by Cochrane handbook for systematic reviews of interventions [16]. Principal authors will be contacted for additional data if necessary.

### Study records

#### Selection process

All titles and abstracts retrieved by searching information sources will be evaluated independently by two authors against the eligibility criteria. If deciding on the eligibility of an article is not possible by screening the title and abstract, the full-text article will be assessed by at least two authors. Disagreements regarding eligibility of studies will be resolved by discussion or consultation with a third researcher when necessary. To avoid duplicate publication bias, we will include only the earliest version of papers that share the same data sets.

#### Data collection process

The following information will be extracted from each included study by two independent authors: bibliographic details: first author, study title, year of publication, and country; demographics and clinical information: number of patients and controls, type of ALS and controls, study design, age, gender, specimen, ALSFRS-R, and duration of disease; measurements: method, value type, and biomarker levels; STROBE-ME checklist [17]. Any disagreements between authors will be resolved by discussion.

#### Risk of bias in individual studies

Two authors will independently assess the quality of included studies and risk of bias using the revised version of Quality Assessment of Diagnostic Accuracy Studies (QUADAS-2) [18]. Summary of quality assessment results and number of papers with a high, low, or unclear risk of bias will be displayed as a table.

We will address the risk of bias due to the effect of motor dysfunction on some cognitive domains (including executive functions, visuoconstruction abilities, psychomotor speed, and MMSE) by effect size re-calculation as was mentioned by Beeldman et al. in their previously published meta-analysis [19].

### Data synthesis

#### Statistical analysis

We will use the mean and standard deviation (SD) of biomarkers concentrations which we extract from individual studies to calculate the standardized mean difference (SMD) and 95% confidence interval (CI). In case of significant heterogeneity, we will use random-effects meta-analysis. Otherwise, the fixed-effects model will be chosen for meta-analysis. All meta-analyses will be performed using the STATA version 14.0.

#### Assessment of heterogeneity

We will apply Q statistic tests and the *I*^2^ index for heterogeneity assessment. The *I*^2^ index will be interpreted according to the Cochrane handbook [16]. When the *I*^2^ index is less than 40%, the statistical heterogeneity is not significant.

#### Investigation of heterogeneity

To find the possible sources of heterogeneity and to investigate their influence on biomarkers levels, we will undertake subgroup analyses based on the following factors if sufficient data are available:

- Population-related factors: type of ALS (sporadic or familial, classic or bulbar-onset), category of ALS diagnosis according to the revised version of El Escorial criteria, co-occurrence of ALS and FTD, type of controls, type of gene mutations, country, race/ethnic group (as is reported in the main article or based on the geographical area of the study), gender, age at the time of diagnosis, survival time, disease severity, and other clinically relevant parameters.
- Intervention-related factors: type of recruitment (population-based or referral-based settings), measurements method (ELISA, Luminex, others)

#### Sensitivity analyses

The influence of methodological quality of included studies on overall effects will be evaluated by excluding studies with high risk of bias and rerunning the analyses.

#### Assessment of reporting bias

We will use the Egger test to assess the influence of unpublished studies or publication bias if the meta-analysis includes a minimum of 5 papers [20].

### Confidence in cumulative evidence

We will use the grading of Recommendations, Assessment, Development and Evaluation (GRADE) approach for assessing the strength of evidence.

## Discussion

In this systematic review and meta-analysis, first, we will assess the concentration of commonly reported biomarkers in patients with ALS, and then, we will clarify how these biomarkers can be related to clinical characteristics of the patients and their disease outcome by subgroup analyses. We hope that the results of this study will help us to characterize ALS and its subtypes better and guide researchers to find possible therapeutic targets and diagnostic tests for ALS.

## Additional files

Additional file 1: PRISMA-P (Preferred Reporting Items for Systematic review and Meta-Analysis Protocols) 2015 checklist. (DOCX 28 kb)

Additional file 2: Search strategy. Keywords that will be used for searching the MEDLINE database. (DOCX 23 kb)

## Abbreviations

ALS: Amyotrophic lateral sclerosis
ALSFRS: ALS functional rating scale
Aβ-42: Amyloid beta
BDNF: Brain-derived neurotrophic factor
FTD: Frontotemporal dementia
GFAP: Glial fibrillary acidic protein
LMN: Lower motor neuron
MCP-1: Monocyte chemoattractant protein-1
MND: Motor neuron disease
PGRN: Progranulin
PRISMA-P: Preferred Reporting Items for Systematic Review and Meta-Analysis Protocols
p-Tau: Phosphorylated tau
QUADAS-2: Quality Assessment of Diagnostic Accuracy Studies
TDP43: TAR DNA-binding protein-43
UMN: Upper motor neuron
