# Supplementary material for: CSF and blood biomarkers in amyotrophic lateral sclerosis: protocol for a systematic review and meta-analysis
Source: Syst Rev. 2018 Dec 20;7:237. doi: 10.1186/s13643-018-0913-4 (PMC6300914; doi:10.1186/s13643-018-0913-4)
Supplement: Supplementary file 2 — Search strategy. Keywords that will be used for searching the MEDLINE database. (DOCX 23 kb) [file 13643_2018_913_MOESM2_ESM.docx]

# Additional file 2

## Search strategy (MEDLINE)

**Aβ42, T-tau, P-tau**

ALS OR MND OR motor-neuron-disease OR motor-neurone-disease OR motor-neurone-diseases OR motor-neuron-disease OR motor-neuron-diseases OR amyotrophic-lateral-sclerosis OR progressive muscular atrophy OR progressive-spinal-muscular-atrophy OR primary-lateral-sclerosis OR motor neuropath* OR motor neuropathies OR lou-gehrig's-disease AND ((("biological"[All Fields] AND "markers"[All Fields]) OR "biomarker"[All Fields]) OR CSF[All Fields] OR ("plasma"[MeSH Terms] OR "plasma"[All Fields]) OR ("serum"[MeSH Terms] OR "serum"[All Fields]) OR cerebrospinal[All Fields]) AND (Aβ42 OR Aβ-42 OR Abeta42 OR Abeta 42 OR Aβ42 OR Abeta42 OR Abeta-42 OR tau1* or tau2* or tau3* or tau4* or tau5* or tau6* or tau7* or tau8* or tau9* OR T-tau OR TTau OR P-tau OR Ptau* OR P-tau* OR phospho-tau OR phosphorylated tau)

**Neurofilaments**

ALS OR MND OR motor-neuron-disease OR motor-neurone-disease OR motor-neurone-diseases OR motor-neuron-disease OR motor-neuron-diseases OR amyotrophic-lateral-sclerosis OR progressive muscular atrophy OR progressive-spinal-muscular-atrophy OR primary-lateral-sclerosis OR motor neuropath* OR motor neuropathies OR lou-gehrig's-disease AND ((("biological"[All Fields] AND "markers"[All Fields]) OR "biomarker"[All Fields]) OR CSF[All Fields] OR ("plasma"[MeSH Terms] OR "plasma"[All Fields]) OR ("serum"[MeSH Terms] OR "serum"[All Fields]) OR cerebrospinal[All Fields]) AND (Neurofilament* OR NEFL OR NFL OR NF-L OR NF68 OR NFH OR pNFH OR pNfH OR phosphorylated neurofilament* OR NF-H OR NFM OR NEFM OR Triplet Proteins OR “heavy neurofilament subunit” OR “neurofilament“)

**S100β**

ALS OR MND OR motor-neuron-disease OR motor-neurone-disease OR motor-neurone-diseases OR motor-neuron-disease OR motor-neuron-diseases OR amyotrophic-lateral-sclerosis OR progressive muscular atrophy OR progressive-spinal-muscular-atrophy OR primary-lateral-sclerosis OR motor neuropath* OR motor neuropathies OR lou-gehrig's-disease AND ((("biological"[All Fields] AND "markers"[All Fields]) OR "biomarker"[All Fields]) OR CSF[All Fields] OR ("plasma"[MeSH Terms] OR "plasma"[All Fields]) OR ("serum"[MeSH Terms] OR "serum"[All Fields]) OR cerebrospinal[All Fields]) AND (S100B OR S100β OR S100beta OR S-100B OR S-100beta OR S-100β)

**Cystatin C**

ALS OR MND OR motor-neuron-disease OR motor-neurone-disease OR motor-neurone-diseases OR motor-neuron-disease OR motor-neuron-diseases OR amyotrophic-lateral-sclerosis OR progressive muscular atrophy OR progressive-spinal-muscular-atrophy OR primary-lateral-sclerosis OR motor neuropath* OR motor neuropathies OR lou-gehrig's-disease AND ((("biological"[All Fields] AND "markers"[All Fields]) OR "biomarker"[All Fields]) OR CSF[All Fields] OR ("plasma"[MeSH Terms] OR "plasma"[All Fields]) OR ("serum"[MeSH Terms] OR "serum"[All Fields]) OR cerebrospinal[All Fields]) AND ( "Cystatin C"[Mesh] OR Cystatin C OR Post-gamma-Globulin OR Post gamma Globulin OR Neuroendocrine Basic Polypeptide OR Cystatin 3 OR gamma-Trace OR gamma Trace)

**PGRN**

ALS OR MND OR motor-neuron-disease OR motor-neurone-disease OR motor-neurone-diseases OR motor-neuron-disease OR motor-neuron-diseases OR amyotrophic-lateral-sclerosis OR progressive muscular atrophy OR progressive-spinal-muscular-atrophy OR primary-lateral-sclerosis OR motor neuropath* OR motor neuropathies OR lou-gehrig's-disease AND ((("biological"[All Fields] AND "markers"[All Fields]) OR "biomarker"[All Fields]) OR CSF[All Fields] OR ("plasma"[MeSH Terms] OR "plasma"[All Fields]) OR ("serum"[MeSH Terms] OR "serum"[All Fields]) OR cerebrospinal[All Fields]) AND (Progranulin OR PGRN)

**GFAP**

ALS OR MND OR motor-neuron-disease OR motor-neurone-disease OR motor-neurone-diseases OR motor-neuron-disease OR motor-neuron-diseases OR amyotrophic-lateral-sclerosis OR progressive muscular atrophy OR progressive-spinal-muscular-atrophy OR primary-lateral-sclerosis OR motor neuropath* OR motor neuropathies OR lou-gehrig's-disease AND ((("biological"[All Fields] AND "markers"[All Fields]) OR "biomarker"[All Fields]) OR CSF[All Fields] OR ("plasma"[MeSH Terms] OR "plasma"[All Fields]) OR ("serum"[MeSH Terms] OR "serum"[All Fields]) OR cerebrospinal[All Fields]) AND (GFAP OR GFA OR "GLIAL FIBRILLARY ACIDIC PROTEIN" OR “GLIAL INTERMEDIATE FILAMENT PROTEIN” OR Astroprotein)

**BDNF**

ALS OR MND OR motor-neuron-disease OR motor-neurone-disease OR motor-neurone-diseases OR motor-neuron-disease OR motor-neuron-diseases OR amyotrophic-lateral-sclerosis OR progressive muscular atrophy OR progressive-spinal-muscular-atrophy OR primary-lateral-sclerosis OR motor neuropath* OR motor neuropathies OR lou-gehrig's-disease AND ((("biological"[All Fields] AND "markers"[All Fields]) OR "biomarker"[All Fields]) OR CSF[All Fields] OR ("plasma"[MeSH Terms] OR "plasma"[All Fields]) OR ("serum"[MeSH Terms] OR "serum"[All Fields]) OR cerebrospinal[All Fields]) AND (Brain?Derived Neurotrophic Factor OR BDNF)

**TDP-43**

ALS OR MND OR motor-neuron-disease OR motor-neurone-disease OR motor-neurone-diseases OR motor-neuron-disease OR motor-neuron-diseases OR amyotrophic-lateral-sclerosis OR progressive muscular atrophy OR progressive-spinal-muscular-atrophy OR primary-lateral-sclerosis OR motor neuropath* OR motor neuropathies OR lou-gehrig's-disease AND ((("biological"[All Fields] AND "markers"[All Fields]) OR "biomarker"[All Fields]) OR CSF[All Fields] OR ("plasma"[MeSH Terms] OR "plasma"[All Fields]) OR ("serum"[MeSH Terms] OR "serum"[All Fields]) OR cerebrospinal[All Fields]) AND (TAR DNA-binding protein 43 OR transactive response DNA binding protein OR TDP-43 OR TDP 43 OR TDP43)

**MCP-1**

ALS OR MND OR motor-neuron-disease OR motor-neurone-disease OR motor-neurone-diseases OR motor-neuron-disease OR motor-neuron-diseases OR amyotrophic-lateral-sclerosis OR progressive muscular atrophy OR progressive-spinal-muscular-atrophy OR primary-lateral-sclerosis OR motor neuropath* OR motor neuropathies OR lou-gehrig's-disease AND ((("biological"[All Fields] AND "markers"[All Fields]) OR "biomarker"[All Fields]) OR CSF[All Fields] OR ("plasma"[MeSH Terms] OR "plasma"[All Fields]) OR ("serum"[MeSH Terms] OR "serum"[All Fields]) OR cerebrospinal[All Fields]) AND (Chemokines CCL2 OR CCL2 OR CCL-2 OR SCYA2 OR MCP1 OR MCP-1 OR MCAF OR "CHEMOKINE CC MOTIF LIGAND 2" OR “SMALL INDUCIBLE CYTOKINE A2” OR “MONOCYTE CHEMOTACTIC PROTEIN 1” OR “MONOCYTE CHEMOTACTIC AND ACTIVATING FACTOR”)

**YKL-40**

ALS OR MND OR motor-neuron-disease OR motor-neurone-disease OR motor-neurone-diseases OR motor-neuron-disease OR motor-neuron-diseases OR amyotrophic-lateral-sclerosis OR progressive muscular atrophy OR progressive-spinal-muscular-atrophy OR primary-lateral-sclerosis OR motor neuropath* OR motor neuropathies OR lou-gehrig's-disease AND ((("biological"[All Fields] AND "markers"[All Fields]) OR "biomarker"[All Fields]) OR CSF[All Fields] OR ("plasma"[MeSH Terms] OR "plasma"[All Fields]) OR ("serum"[MeSH Terms] OR "serum"[All Fields]) OR cerebrospinal[All Fields]) AND (CHIT? OR chitotriosidase OR CHI3L1 OR GP39 OR YKL40 OR YKL-40 OR YKL 40 OR Chondrex OR cartilage gp-39 OR "CHITINASE 3-LIKE 1" OR "CARTILAGE GLYCOPROTEIN 39")

**CHIT1**

ALS OR MND OR motor-neuron-disease OR motor-neurone-disease OR motor-neurone-diseases OR motor-neuron-disease OR motor-neuron-diseases OR amyotrophic-lateral-sclerosis OR progressive muscular atrophy OR progressive-spinal-muscular-atrophy OR primary-lateral-sclerosis OR motor neuropath* OR motor neuropathies OR lou-gehrig's-disease AND ((("biological"[All Fields] AND "markers"[All Fields]) OR "biomarker"[All Fields]) OR CSF[All Fields] OR ("plasma"[MeSH Terms] OR "plasma"[All Fields]) OR ("serum"[MeSH Terms] OR "serum"[All Fields]) OR cerebrospinal[All Fields]) AND (chitotriosidase OR CHIT1 OR CHIT 1 OR CHIT-1 OR CHIT?)
